# Open Targets Platform: new developments and updates two years on

**DOI:** 10.1093/nar/gky1133

**Published:** 2018-11-20

**Authors:** Denise Carvalho-Silva, Andrea Pierleoni, Miguel Pignatelli, ChuangKee Ong, Luca Fumis, Nikiforos Karamanis, Miguel Carmona, Adam Faulconbridge, Andrew Hercules, Elaine McAuley, Alfredo Miranda, Gareth Peat, Michaela Spitzer, Jeffrey Barrett, David G Hulcoop, Eliseo Papa, Gautier Koscielny, Ian Dunham

**Affiliations:** 1 European Molecular Biology Laboratory, European Bioinformatics Institute (EMBL-EBI), Wellcome Genome Campus, Hinxton, Cambridgeshire CB10 1SD, UK; 2 Open Targets, Wellcome Genome Campus, Hinxton, Cambridgeshire CB10 1SD, UK; 3 Wellcome Sanger Institute, Wellcome Genome Campus, Hinxton, Cambridge CB10 1SA, UK; 4 GSK, Medicines Research Center, Gunnels Wood Road, Stevenage, SG1 2NY, UK; 5 Biogen, Cambridge, MA 02142, USA

## Abstract

The Open Targets Platform integrates evidence from genetics, genomics, transcriptomics, drugs, animal models and scientific literature to score and rank target-disease associations for drug target identification. The associations are displayed in an intuitive user interface (https://www.targetvalidation.org), and are available through a REST-API (https://api.opentargets.io/v3/platform/docs/swagger-ui) and a bulk download (https://www.targetvalidation.org/downloads/data). In addition to target-disease associations, we also aggregate and display data at the target and disease levels to aid target prioritisation. Since our first publication two years ago, we have made eight releases, added new data sources for target-disease associations, started including causal genetic variants from non genome-wide targeted arrays, added new target and disease annotations, launched new visualisations and improved existing ones and released a new web tool for batch search of up to 200 targets. We have a new URL for the Open Targets Platform REST-API, new REST endpoints and also removed the need for authorisation for API fair use. Here, we present the latest developments of the Open Targets Platform, expanding the evidence and target-disease associations with new and improved data sources, refining data quality, enhancing website usability, and increasing our user base with our training workshops, user support, social media and bioinformatics forum engagement.

## INTRODUCTION

Drug discovery is a long and costly endeavour characterized by high failure rates. Failure often occurs at the later stages of the drug discovery pipeline and the reasons for the low success are largely twofold: lack of safety and/or lack of efficacy. This reflects insufficient understanding of the role of the chosen target in disease, and the consequences of modulating it with a drug. Over the last several years, there has been an increase in the number of biological and chemical databases available for better understanding of drug targets (1). These databases can be used to assist with target identification, one of the most important stages in drug discovery (2).

The Open Targets Platform (https://www.targetvalidation.org) is a freely available resource for the integration of genetics, omics and chemical data to aid systematic drug target identification and prioritisation. The Open Targets Platform capitalises on publicly available databases to create a virtuous cycle where we add value to the original data by computing, scoring and ranking integrated target-disease associations (3), linking these associations back to the underlying evidence and its provenance.

We have expanded the Platform to include data from more projects and initiatives in translational research and medicinal chemistry, such as Genomics England (4), the Structural Genomics Consortium (https://www.thesgc.org) and the Institute of Cancer Research (https://www.icr.ac.uk), and continue to adhere and contribute to international naming standards and ontologies through our ongoing collaboration with the Experimental Factor Ontology (EFO) (5) and the Evidence & Conclusion Ontology (ECO) (6).

Although the main port of access for all our data is a graphical user interface (GUI) designed for bench scientists working in early drug discovery (7), we have observed an uptake in the use of our REST-API and data downloads. Moreover, due to the availability of the Open Targets Platform snapshots, our database can now be re-created by other parties. Here, we describe the developments since our first publication, focusing on the new data sources for target-disease associations, new target and disease annotations for target prioritisation, and new intuitive visualisations designed with ongoing focus on usability.

## NEW DEVELOPMENTS AND PROGRESS

### More data with continuing emphasis on user experience

A key factor for drug target identification and prioritisation is the causal association of the target with a disease. We compute an association score based on genetics, genomics, transcriptomics, drug information, animal models and scientific literature evidence. Our scoring framework has been described in our previous publication (3). Briefly, the computation is carried out at four different levels to give rise to evidence scores, data source scores, data type scores, and an overall association score. To compute the evidence score, we take into account specific factors that affect the strength of the evidence used for the target-disease associations (See table 2 in (3). In order to obtain a score for data sources and data types, we use the harmonic sum to aggregate individual evidence and data source scores, respectively. Our overall association score is the result of the aggregation of all data sources using the harmonic sum (Supplementary Figure S1).

Since our first publication, we have continued to explore new datasets to be included as evidence for new target-disease associations or refinement of existing ones. Our criteria to consider new data sources are: (i) relevance (can the data be used to associate targets with diseases? Does it suggest a causal link between a target and a disease? Does it enable prioritisation decision by target properties?); (ii) ease of integration (does the data use an ontology? Are the targets provided as either UniProt ID or Ensembl gene IDs? How much term mapping will be required? Is there a score or threshold that can be used to rank the data points?); (iii) accessibility (is the data publicly available, free and easy to access through an API or downloads?) and (iv) sustainability (is the data source likely to be maintained over the long term? Is the data frequently updated?). Once we select new data sources, they are combined into broader data types: Genetic associations, Somatic mutations, Drugs, Affected pathways, RNA expression, Animal models and Text mining.

In addition to including new data sources since our first publication (3), we have carried out further quality assessment of our transcriptomics evidence and expanded the scope and coverage of many of our original data sources.

### New data sources for target-disease associations

We have incorporated four new data sources to enhance our evidence: Genomics England PanelApp and the PheWAS catalogue (within the data type Genetic associations) and SLAPenrich and PROGENy (for the data type Affected pathways).

### Genetic associations

Since our previous publication, we have added two new data sources as evidence for Genetic associations between targets and Mendelian and more common diseases: Genomics England PanelAPP and the PheWAS catalog, respectively. With these new data sources, we have been able to identify new associations (e.g. between SERPING1 and Immunodeficiency due to an early component of complement deficiency based on evidence from the Genomics England PanelAPP, or between MC1R in hyperlipidemia based on evidence from the PheWAS catalog) or added further support to previously identified associations (e.g. between KCNE3 in Brugada syndrome based on evidence from the Genomics England PanelAPP, or NOD2 in Crohn's disease based on evidence from the PheWAS catalog).

We have included the Genomics England PanelApp Green genes (version 1+ panels) (4) along with their (mainly) rare, Mendelian diseases or phenotypes, providing these can be mapped to an ontology, such as EFO (5), Orphanet (http://www.orpha.net) or Human Phenotype Ontology (HP) (8). We use the PanelApp WebServices (https://panelapp.genomicsengland.co.uk/#!Webservices) to obtain the associations and Ontoma (https://pypi.org/project/ontoma/) for the automatic mapping of diseases and phenotypes.

The Genomics England Green genes are curated and crowdsourced by experts; hence the target-disease associations that are supported by this evidence in our Platform have the highest score of 1.

For common and complex diseases, we have added genetic evidence from PheWAS (9), scored following our methodology for GWAS evidence (3) but scaled according to the maximum number of cases (8800) and the *P*-value range (0.05 and 1e-25) of the PheWAS data.

Details on the scoring of these new data sources for Genetic associations are described in our help documentation (https://docs.targetvalidation.org/getting-started/scoring).

### Affected pathways

Besides the new data sources for Genetic associations, we have also included two new data sources for Affected pathways, more specifically in cancer. The new data sources, SLAPenrich (10) and PROGENy (11), have mostly highlighted new associations, such as EGFR in squamous cell lung carcinoma based on PROGENy and PTEN in prostate adenocarcinoma based on SLAPenrich.

SLAPenrich identifies pathways that harbour genomic alterations, more frequently than expected by chance, across a population of cancer samples from their somatic mutation profiles. The alteration status of a pathway is determined by the collective status of its genes: a pathway is altered in a sample if at least one of its genes is somatically mutated in that sample. Then, SLAPenrich quantifies the divergence from expectation of the total number of samples with genomic alterations in a pathway, using a Poisson binomial distribution. The SLAPenrich evidence used for the associations between targets and cancer is based on Reactome-pathway gene-sets only, diverging from the original analysis (10).

PROGENy (11) is a method to infer pathway activity from gene expression. The data includes 11 pathways, namely EGFR (epidermal growth factor receptor), MAPK (mitogen-activated protein kinase), PI3K (phosphoinositide 3-kinase), VEGF (vascular endothelial growth factor), JAK-STAT (janus kinase-signal transducer and activator of transcription), TGFb (transforming growth factor beta), TNFa (tumor necrosis factor alpha), NFkB (nuclear factor kappa-light-chain-enhancer of activated B cells), Hypoxia, p53-signaling and DNA damage response, and cell death via apoptosis (Trail). In contrast to other pathway methods, PROGENy is based on the gene expression signature downstream of the pathway, rather than on the expression of the pathway components, generating signatures that represent a consensus over many different conditions. Contrary to SLAPenrich, which implicates pathways but not their activation or inhibition, PROGENy scores indicate whether a pathway is activated or inhibited if they are higher or lower between conditions. Both tools can hence be used in combination to infer driver pathways hit by mutations, and then link those to their downstream effects.

Details on the scoring of these new data sources for Affected pathways are described in our help documentation (https://docs.targetvalidation.org/getting-started/scoring).

### Updated data sources for disease and target associations

In addition to new data sources, since our first publication we have also had updates on the original data sources through our frequent cycles of data release. Our data ingest and processing pipeline is now run every two months for the integration of the most up-to-date information from our data providers. Each release has a YY.MM timestamp and may contain increased (and updated) coverage of the evidence described in our original paper (3) as well as new data sources (see above section). Furthermore, some of the original data sources have had their scope expanded or amended through ongoing projects within Open Targets. These correspond to the (i) inclusion of targeted genotyping arrays from the GWAS Catalogue (12); (ii) addition of the new tier 2 cancer genes (13) from the Cancer Gene Census (14); (iii) coverage of trinucleotide repeat data from ClinVar (15) available in the European Variation Archive (https://www.ebi.ac.uk/eva/?Home) and (iv) withdrawal of differential expression studies reported on human cell lines with no disease as study factor from Expression Atlas (16). In the following sections, we describe the updates in the original data sources used for our target-disease associations in more detail.

### Targeted, non genome-wide genotyping arrays

We have started a collaboration with the NHGRI-EBI GWAS Catalogue (12) for the inclusion of non genome-wide arrays including the Immunochip (17) for immunogenetics, and Metabochip (18) for metabolic diseases. So far, this has enabled us to include 823 SNP-trait associations for 120 independent associations curated from 55 publications. The inclusion of both Immunochip and Metabochip arrays increases the availability of germline variants (or SNPs) that are associated with autoimmune, inflammatory and metabolic diseases. These causal genetic variants in the Open Targets Platform will help uncover strong candidate genes for those diseases, prioritise target-disease associations and explore pleiotropy, if the candidate genes are associated with more than one of the diseases for which the array was designed.

### Tiered cancer gene census

In our initial paper (3), we described somatic mutation evidence from the Cancer Gene Census (14) used to support target-cancer associations. This census has recently introduced new criteria to assess the level of evidence that supports a gene as a driver gene in cancer, which leads to the concept of a tier system (13). Genes in tier 1 must have: (i) evidence of activity that may drive or suppress cancer; (ii) evidence of mutations, detected in cancer that change the activity of the protein and promote oncogenic transformation and (iii) evidence that the somatic mutation patterns in cancer samples are typical of tumour suppressor genes (e.g. inactivating mutations) or of oncogenes (e.g. missense mutations). Although tier 2 genes are strongly associated with a role in cancer, they have less evidence than their tier 1 counterparts. For both tier 1 and 2 genes, Poisson tests are carried out to assess whether somatic mutations in a gene occur more frequently than in other genes in the same disease, and whether a gene is mutated significantly more frequently in a given disease when compared to all other diseases. All mutations detected in the Cancer Gene Census genes have a base association score of 0.5, which is modified by applying the following rules: (i) for tier 1 genes, a significant result (FDR < 0.025) for either of each of the two Poisson tests adds 0.25 to the score; (ii) for tier 2 genes, the score will be 0.5; (iii) if only one sample is mutated, 0.25 is subtracted from the score and (iv) if tier 1 genes are known to drive cancer only through fusions, all mutation types except fusions get a score of 0.5. Fusions will then be scored according to the rules above.

### Trinucleotide repeat expansions and new clinical significance terms

We import trinucleotide repeats from ClinVar (15) that are stored in the European Variation Archive (https://www.ebi.ac.uk/eva/?Home) for the genetic associations between triplet repeat expansion disorders, e.g. Huntington’s Disease and Fragile X syndrome, and their possible drug targets, such as Huntingtin and Synaptic functional regulator FMR1 proteins. In order to incorporate trinucleotide repeats, a new consequence term, trinucleotide expansion (SO_0002165) (https://www.targetvalidation.org/variants) was defined by ECO (6). Moreover, besides mutations described as ‘pathogenic’, which have been included since the first release of the Open Targets Platform, we have begun incorporating mutation data with other terms of clinical significance from ClinVar, namely ‘protective’, ‘association’, ‘risk factor’, ‘affects’ and ‘drug response’. The full description of these terms are available elsewhere (https://www.ncbi.nlm.nih.gov/clinvar/docs/clinsig/).

### Human cell lines

We have removed studies from Expression Atlas where experiments were carried out in human cell lines where the disease was not a factor in the study (e.g. cell lines derived from cancers used in other studies). This has lead to a 34% reduction in the number of evidence strings for the assessment of differential expression of drug targets, and therefore removal of false-positive associations in the RNA expression data type.

In summary, new data sources, quality assessment and further refinements to the original set of data sources have increased the scope of our target-disease associations. A summary of the latest set of data sources and count of evidence from each source are provided in Table 1. The statistics for our releases (current and previous ones) can be found in our Release Notes (https://www.targetvalidation.org/release-notes).

**Table 1.** Sources and evidence counts used for target-disease associations in the Open Targets Platform

Data source*	Data type	Evidence Count**
**Genomics England PanelAPP (v2.2.0)**	Genetic associations	15 289
**PheWAS catalogue (Sep-2017)**	Genetic associations	47 302
GWAS catalogue (July 2018)	Genetic associations	101 511 (*32 363*)
UniProt (July 2018)	Genetic associations	26 640 (*21 870*)
UniProt literature (July 2018)	Genetic associations	4494
European Variation Archive∧ (July 2018)	Genetic associations	73 805 (*28 050*)
Gene2Phenotype (May 2017)	Genetic associations	1604 (*975*)
UniProt (July 2018)	Somatic mutations	282
Cancer Gene Census (COSMIC v85)	Somatic mutations	55 963 (*23 440*)
IntOGen (December 2014)	Somatic mutations	2371 (*2377*)
European Variation Archive∧ (July 2018)	Somatic mutations	7624 (*456*)
ChEMBL (v24)	Drugs	410 436 (*120 520*)
Reactome (v65)	Affected pathways	9735 (*6143*)
**PROGENy (April 2018)**	Affected pathways	308
**SLAPenrich (August 2017)**	Affected pathways	89 661
Expression Atlas (February 2018)	Expression	288 273 (*529 084*)
Europe PMC (July 2018)	Text mining	4 906 527 (*3 678 967*)
PhenoDigm (November 2017)	Animal model	465 887 (*395 331*)

*Database version (or date) in parentheses.

**As per 18.08 release of the Open Targets Platform. Parentheses show the number (in italics) of evidence count reported previously (3). Note, the reduction in the number of evidence from Expression Atlas (see main text for explanation).

∧Containing ClinVar data from May 2017.

Detailed target-disease association counts can be found in the Supplementary Table.

Data sources in bold are new data, whereas the remaining sources have been described in our first publication and shown here are updates from the previous report.

### New annotations and visualisations for targets and diseases

Besides providing target-disease associations, the Open Targets Platform integrates comprehensive annotation of individual human targets and diseases on dedicated pages to support target prioritisation. The target profile page contains information at the gene and protein levels, whereas the disease profile page displays disease annotations, such as phenotypes and ontology classification. Note that our targets can be both protein coding genes and non-coding RNA genes, such as HOTAIR and MIR23A non-coding genes.

Since our first publication, target and disease annotations have been enhanced with new visualisations and data. A new plugin architecture for the pages enables widgets and/or data to be added more easily, allowing increased content flexibility and faster loading time for a better user experience. We have also changed the order of the annotations displayed in the target profile page based on usage statistics from anonymised web traffic logs, now displaying the more relevant information first, at the top of the page. In the following sections, we provide details on these new target annotations, in addition to updates on the visualisation of expression data and scientific literature.

### Target enabling packages and chemical probes

A Target Enabling Package (TEPs) is a collection of reagents, protocols and data for rapid exploration and characterization of proteins (potential drug target candidates) with genetic linkage to key disease areas (19). All 16 TEPs, currently available from the Structural Genomics Consortium portal, can be accessed from the relevant target profile pages in the Open Targets Platform.

We also link to a set of the 215 high-quality chemical probes (20,21) available for 188 different targets, giving access to reagents and assays to aid *in vitro* and/or *in vivo* investigation of phenotype and mechanism of a target. An additional set of potential chemical probes for 2300 human targets from Probe Miner (https://probeminer.icr.ac.uk/#/) is also available in the Open Targets Platform.

### Protein–protein interactions

**Figure 1.**
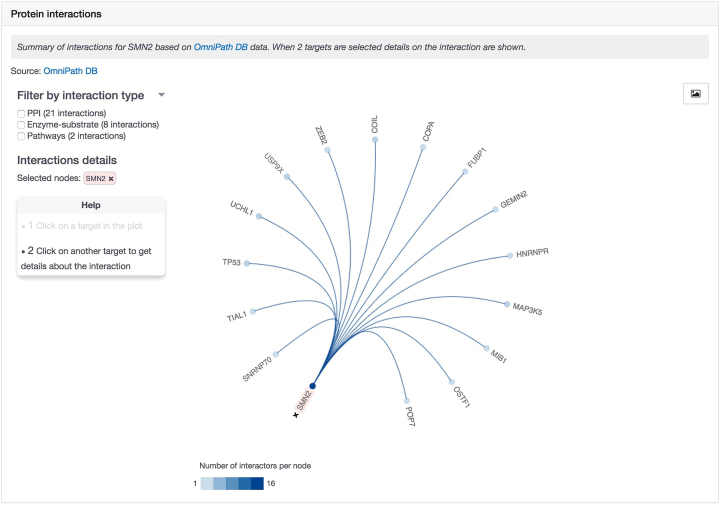
Interactive visualisation of protein–protein interactions in dedicated target profile pages.

We provide a summary of direct protein interactions with the selected target to explore interactome information and facilitate drug target prioritisation. Currently, we display a summary of protein interaction data from OmniPath (22). This data can be filtered by enzyme–substrate interactions, protein–protein interactions or pathways (Figure 1).

### Mouse phenotypes

We list the annotated strain-specific phenotypes and the allelic composition of every laboratory mouse with a knockout gene curated by and available in the Mouse Genome Informatics database (23).

### Cancer hallmarks

We summarise information on cancer hallmarks (24,25), which are curated by COSMIC (26) and integrated in the Cancer Gene Census (14). These essential alterations in cell physiology that can dictate malignant growth can be found in the profile pages of a target implicated in cancer. We list the hallmarks (e.g. invasion and metastasis, change of cellular energetics) for a given target as either being promoted or suppressed. We currently have cancer hallmarks for 251 targets from the Cancer Gene Census.

### Cancer biomarkers

We incorporate a collection of genomic biomarkers of drug responses (sensitivity, resistance and toxicity) and their level of clinical significance from the Cancer Biomarkers database (27).

### Similar targets and their diseases

We have developed a new feature to show suggested targets that are similar to any target of choice. This is based on a network analysis of shared diseases obtained from our set of target-disease associations as a bipartite graph, with targets and diseases as vertices. In order to reduce noise in the data, we consider only disease-association pairs with at least three evidence supporting the edge, and whose overall association score is greater than 0.1. We calculate a target relationship score based on the ratio of shared diseases between the targets to the total number of diseases for both targets.

**Figure 2.**
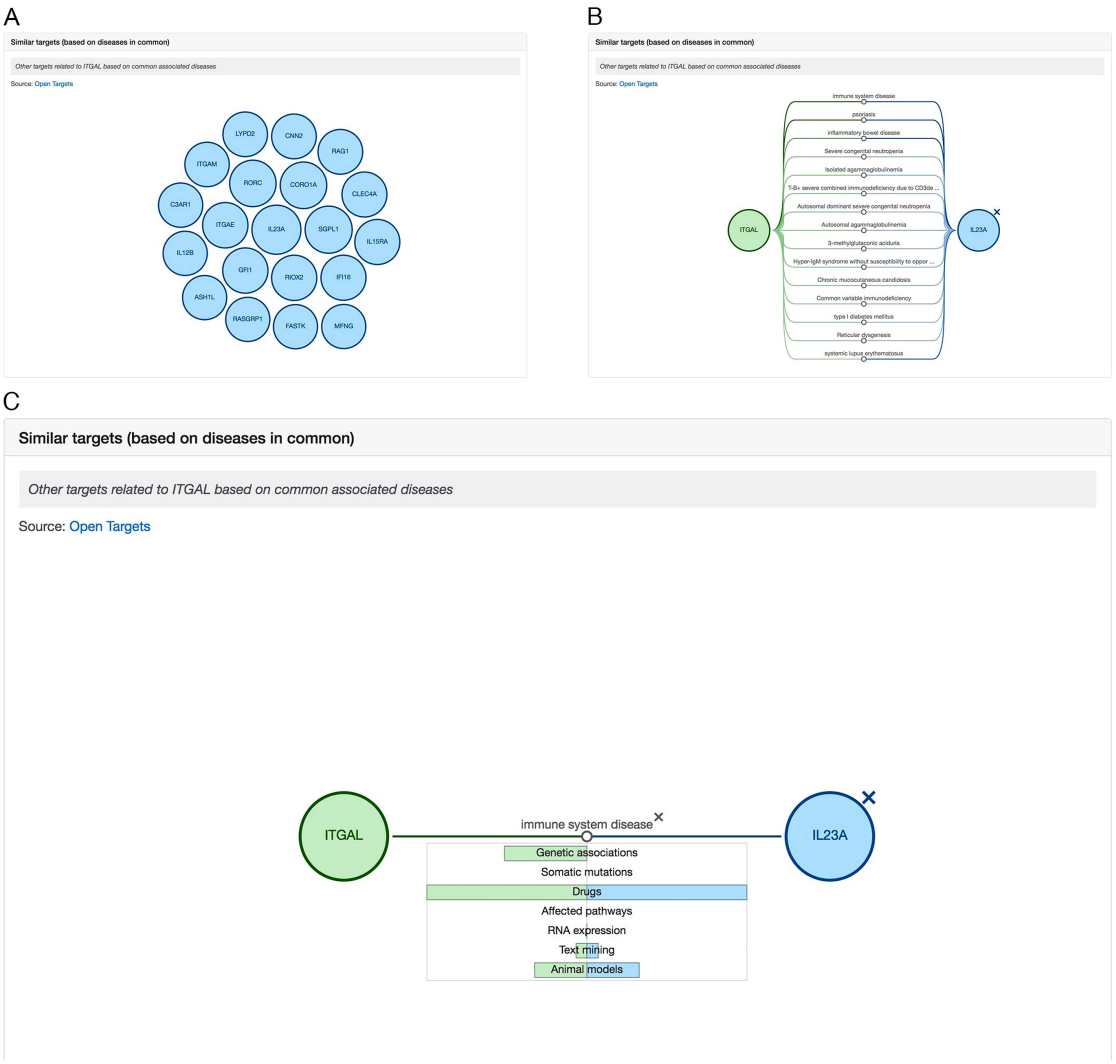
Similar targets are displayed as an interactive visualisation (**A**) in the target profile page. By selecting a target, the view gets updated to show the diseases shared between any two targets (**B**). Clicking on any of the shared diseases reveals the underlying evidence (e.g. Genetic associations, Drugs, Text mining, Animal models) that supports the association between a disease and its two selected targets.

Our relationship scoring method (i) outputs a closer distance between two targets sharing a rare disease than two targets sharing diseases that are data rich, such as cancer and (ii) considers shared targets that are specifically linked to fewer diseases more relevant than targets that are commonly linked to many types of diseases. In order for this process to remain computationally feasible, given the billions of possible target–target (and disease–disease) combinations, we have implemented an efficient, high performance computation strategy, which uses (i) an heuristic estimation from aggregate statistics allowing us to skip the computation of the distance for pairs that are below the cut-off and (ii) LSH (locality-sensitive hashing) (28–30) to calculate target relatedness, retaining only the most confident relationships. The resulting set of similar targets are displayed as an interactive visualisation (Figure 2A), which summarises the top ranking shared diseases for any two targets up to 20 (Figure 2B), and presents the underlying evidence (e.g. Genetic associations, Affected pathways, Drugs) supporting the association (Figure 2C).

The relationship scoring procedure is also applied for diseases sharing the same targets and the relations can be visualised on the disease profile page.

### RNA and protein baseline expression

**Figure 3.**
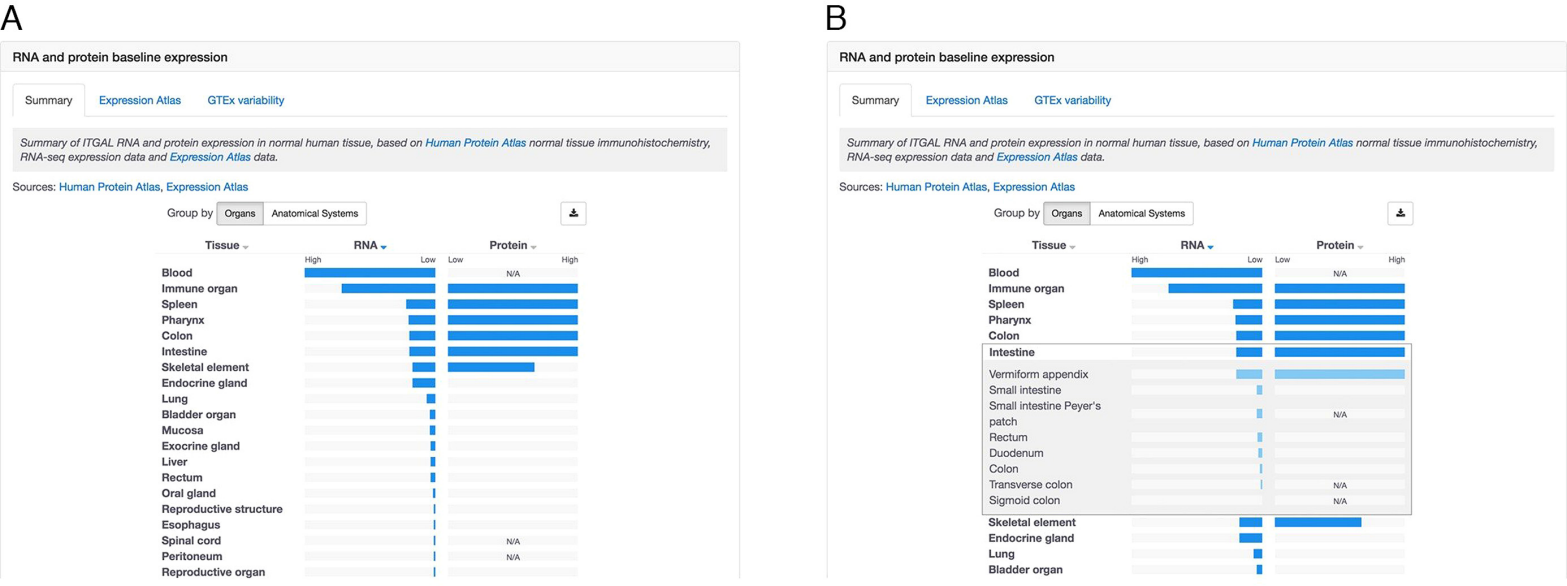
RNA and protein expression data are displayed side by side for easy comparison of target expression levels in healthy human tissues. (**A**) Each horizontal bar representing a tissue, e.g. Intestine, can be expanded to provide a detailed breakdown of expression in different parts of the tissue/organ, such as ‘Vermiform appendix’ and ‘Duodenum’ (**B**).

**Figure 4.**
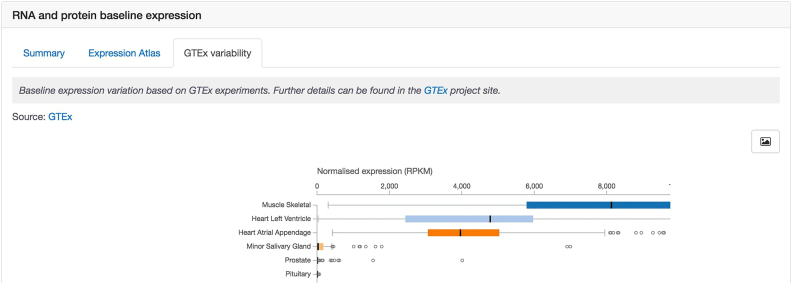
An additional visualisation to summarise expression data is also available, depicting gene expression variability in GTEx data.

When trying to identify a new target, users working in drug discovery often want to understand the expression of the target across human tissues and cells. We have enhanced both the data and the visualisation for baseline expression by combining both RNA (16) and protein (31) expression data under a single section entitled ‘RNA and protein baseline expression’. Within this section, there are three tabs, ‘Summary’, ‘Expression Atlas’ and ‘GTEx variability’. The first tab shows RNA and protein expression data side by side for a quicker comparison (Figure 3A). This can be especially useful for targets that show different levels of RNA and protein expression, e.g. low expression at the RNA level, but high expression at the protein level. The expression data can be visualised grouped either by ‘Organs’ (by default) or ‘Anatomical Systems’. For either option, users can click on the name of a tissue, e.g. ‘Intestine’, and see a detailed breakdown of expression in different parts of the tissue/organ, such as ‘Vermiform appendix’ and ‘Duodenum’ (Figure 3B). Two other displays of expression data are also available in additional tabs: an interactive heat map from Expression Atlas (16) and a box plot to visualise gene expression variability in GTEx data (32) (Figure 4).

### Bibliography

**Figure 5.**
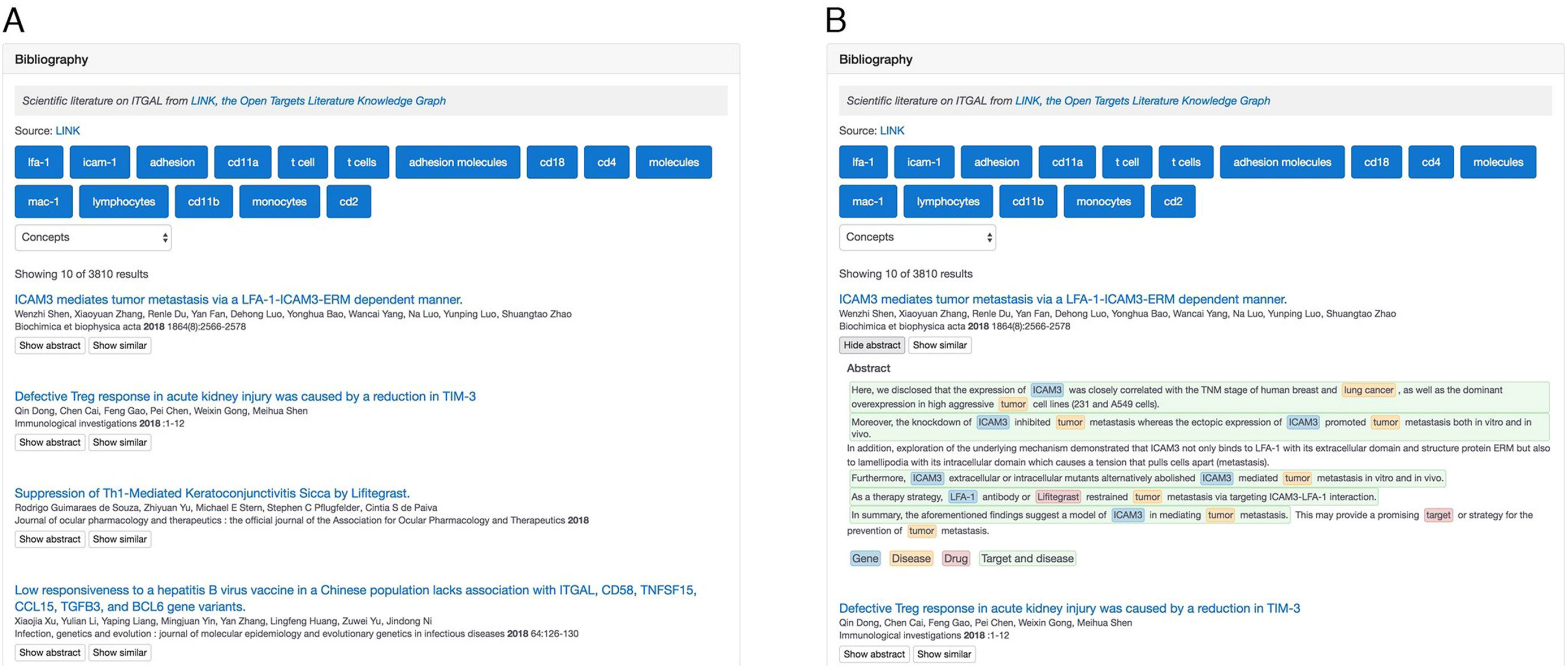
The new visualisation in the ‘Bibliography’ section of target and disease profile pages. Both titles (**A**) and abstracts (**B**) are available and can be filtered by selecting one of the ‘chips’ at the top of the table. A drop-down menu is also available to allow selection of publications according to the available (biological) concepts, genes, diseases, drugs, journals and authors.

We have improved how the ‘Bibliography’ section is displayed on both target and disease profile pages. We have moved to a bespoke navigation and visualisation of scientific papers from Europe PMC (33) using ‘chips’ (Figure 5A). These are created from automated topic identification and entity recognition using LINK, the Open Targets LIterature coNcept Knowledgebase (https://link.opentargets.io). LINK extracts key entities from PubMed abstracts (34) using a precompiled set of dictionaries to recognise genes and diseases from the Open Targets Platform, phenotypes from the Human Phenotype Ontology (8), drugs in clinical trials or on the market from ChEMBL (35), and relevant MESH headings, such as anatomy, diagnostics and locations. We analyse each title and abstract with spaCY (36), and extract key concepts and semantic relations in the form of subject-predicate-object triples.

A drop-down menu is also available in the ‘Bibliography’ section to filter the publications according to key entities, i.e. concepts (e.g. loss of heterozygosity), genes, diseases, drugs, journals and authors. Both drop down menu and ‘chips’ allow for interactive filtering of abstracts (Figure 5B) selected in the target and disease profile pages of the Open Targets Platform.

### New filtering options in the user interface and URL sharing

We have introduced filters on the GUI to allow for targets to be selected based solely on their properties and facilitate prioritisation of the most promising (best) targets for downstream analysis. The properties available for filtering are ‘Target class’, e.g. enzyme, surface antigen, as defined by ChEMBL (35) and ‘RNA tissue specificity’, e.g. to restrict targets that are expressed preferentially in the selected cell or tissue (e.g. brain) compared to other tissues, based on Expression Atlas data (16). We have also implemented ‘Your target list’, where users can upload their own list (in .CSV or .TXT) of targets (either as Ensembl gene IDs, HGNC symbols, UniProt IDs or synonyms) to restrict the associations table to the user’s targets only.

Other changes to the interface include URL sharing for specific views and pages, such as the bubbles view (‘?view=t:bubbles’ in the URL) and the evidence page based on a specific type of data e.g. Genetic associations (‘view=sec:genetic_association’ in the URL).

### Alternative ways to access data

The user interface of the Open Targets Platform allows searches for an entry to be carried out on a one-by-one case basis only: one disease or one target, for example. However, we have use cases that start from a list of targets, rather than a single target. For bulk searching, we have launched a batch search at https://www.targetvalidation.org/batch-search, an easy-to-use and interactive web tool that takes a list of up to 200 targets, identified by HGNC symbols, UniProt or Ensembl IDs, or gene/protein synonyms, and uploaded as .TXT or .CSV. In addition to upload, users can also paste their targets into the box available in the entry page. The batch search will return (i) diseases associated with the list of targets ranked by significance using a hypergeometric distribution; (ii) pathways enriched in the set of targets ranked by the probability of finding a pathway that is associated with and specific to the target list; (iii) gene ontology terms enriched among the targets, ranked following a hypergeometric distribution; (iv) drugs that are known to modulate the targets in the list and (v) a visualisation of protein interactions, showing the interactions between the targets in the batch list. From the batch search results, links to other pages in the Open Targets Platform are available for further exploration of pathway and drug summaries, and/or associations and evidence pages. A detailed tutorial on the batch search tool is available in our documentation page (https://docs.targetvalidation.org/getting-started/batch-search).

The Open Targets REST API provides extra flexibility for data retrieval and filtering options for larger queries, which are not supported by the batch search. The REST API has been updated to version 3, changed its base URL to https://api.opentargets.io/v3/platform and no longer requires an API key for fair usage. More details on the REST API can be found in our documentation page (https://api.opentargets.io/v3/platform/docs/swagger-ui), an interactive page where users can execute the REST API calls and check the response before using these calls in workflows for automated analysis.

### New name, new homepage and improved search functionality

We have renamed the portal from ‘Target Validation Platform’ to ‘Open Targets Platform’ in line with the rebranding of the overall partnership (http://www.opentargets.org). The homepage has been completely redesigned to highlight the main entry point for our users, the search function. In addition to target and disease names (or symbols and their synonyms), we allow searching for phenotypes, orthologous genes (e.g. from mouse, rat, fruit fly, worm) and drugs. If searching for a drug, the Platform accepts chemical (e.g. acetylsalicylic acid), generic (aspirin) and brand (Durlaza) names, and will return a list of targets and diseases that have associations where the drug is involved. When searching for drug names, these get matched to the ‘drugs.evidence data’ field, which contains words, such as Acetylsalicylic acid, Acetylsalicylic Acid, acetylsalic acid, Salicylic Acid Acetate, Acetylsalic Acid, Durlaza.

The new homepage displays the main statistics of the latest release of the Open Targets Platform, namely the number of targets, diseases, associations and data sources that provide evidence for the target-disease associations. It also provides links to other modes of data access (see above), directs users to free tutorials and documentation pages, and includes feeds from Open Targets social media channels, such as the Blog, Twitter and Facebook.

### Outreach, training and user support

The GUI https://www.targetvalidation.org is the main portal to access data from the Open Targets Platform. It had 685 visits and 2369 unique page views from 45 countries in the week prior to the submission of our first publication, i.e. between 12 August 2016 and 19 August 2016. We have observed an increase to 1290 visits and 5411 unique page views from 62 countries for the same eight-day period in 2018 (12 August to 19 August). We offer free hands-on workshops on the Open Targets Platform, both face-to-face and as live webinars (Carvalho-Silva *et al.* DOI: 10.1371/journal.pcbi.1006419). Our recorded webinars, online tutorials and short demos are available on the Open Targets YouTube channel (https://www.youtube.com/channel/UCLMrondxbT0DIGx5nGOSYOQ/featured), which features 12 videos. Our user community can follow our news and upcoming developments on Twitter (twitter.com/targetvalidate), Facebook (https://www.facebook.com/OpenTargets/), LinkedIn (https://www.linkedin.com/company/open-targets/), and by subscribing to our monthly newsletters (http://bit.ly/Open-Targets-News). We also have a blog (http://blog.opentargets.org) that featured 17 posts over the last 12 months and is mirrored on Medium (https://medium.com/opentargets). Direct support via email is available through support@targetvalidation.org.

## CONCLUSIONS

The Open Targets Platform is part of an increasing effort on the integration of public resources to assist target identification and prioritisation for drug discovery. Although many of these resources focus on interactions between drug compounds and their targets, fewer databases explore the evidence available that links a target with a disease. Related resources to the Open Targets Platform, such as DisGeNET (37,38) and Pharos (39,40), have been compared and their complementarities and differences highlighted in our previous publication (3). Recently, Zhang *et al* (1) have provided a more comprehensive comparative analysis.

Following its launch in December 2015, the Open Targets Platform has been expanding its scope, increasing its data coverage, reaching out to user communities worldwide, and growing its user base, all carried out with a constant focus on usability and user design to maintain its easy-to-use and interactive features. We currently integrate over six billion evidence from 18 publicly available data sources and compute almost three billion associations between 21 149 human genes and 10 101 disease and phenotypes. The upcoming months will see the integration of new data from the Open Targets experimental programme including synthetic lethality data from CRISPR/Cas9 knockout screens in cancer cell lines, as well as the release of Open Targets Genetics (https://genetics.opentargets.org/), a new resource that combines GWAS and functional genomics data to prioritise likely causal variants at disease-associated loci. In summary, we will sustain and build on our efforts to date, and continue to provide the Open Targets Platform to facilitate drug target identification and prioritisation, and ultimately increase the odds of success in drug discovery.

## Supplementary Material

Supplementary Data
