# Supplementary material for: Open Targets Platform: new developments and updates two years on
Source: Nucleic Acids Res. 2018 Nov 20;47(Database issue):D1056–65. doi: 10.1093/nar/gky1133 (PMC6324073; doi:10.1093/nar/gky1133)
Supplement: Supplementary Data [file gky1133_supplemental_files.pdf]

Supplementary Figure 1: Schematic representation of the scoring framework for our target-disease associations

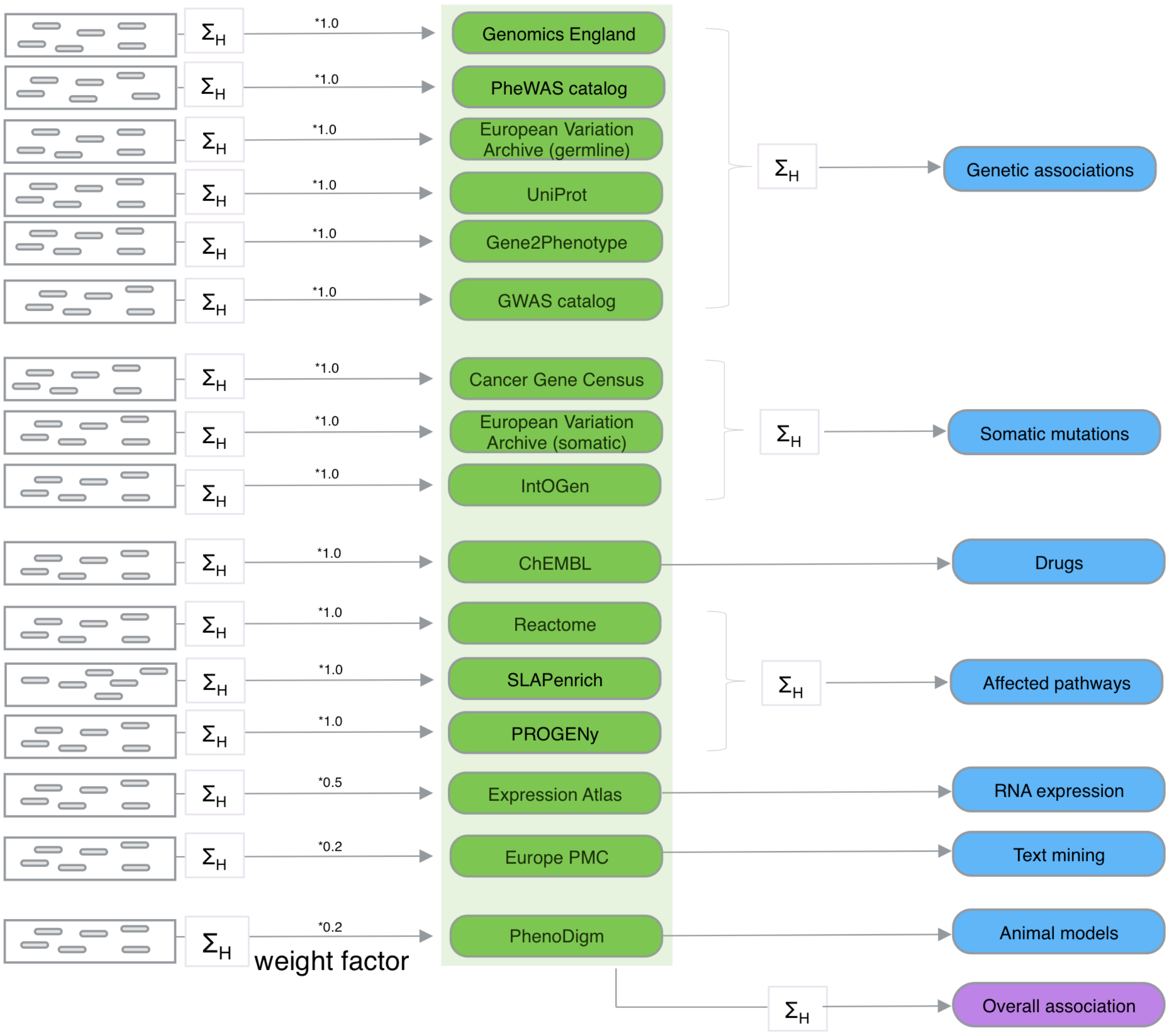

Target-disease associations are ranked and prioritise based on the scores calculated at four different levels: evidence score (in white), data source score (in green), data type score (in blue), and overall association score (in purple). The evidence score is obtained with the formula $f * s * c$, with the factors affecting the relative strength of the evidence differing across the different data sources. Details on our scoring framework have been published elsewhere (3) and its current format is available on https://docs.targetvalidation.org/getting-started/scoring.

Supplementary Table 1: Data sources, data types and weight factor for the association score

Data source	Data type	Weight factor
**Genomics England PanelAPP**	Genetic associations	x1.0
**PheWAS catalog**	Genetic associations	x1.0
GWAS catalog	Genetic associations	x1.0
UniProt**	Genetic associations	x1.0
European Variation Archive (germline)	Genetic associations	x1.0
Gene2Phenotype	Genetic associations	x1.0
Cancer Gene Census	Somatic mutations	x1.0
IntOGen	Somatic mutations	x1.0

European Variation Archive (somatic)	Somatic mutations	x1.0
ChEMBL	Drugs	x1.0
Reactome (v65)	Affected pathways	x1.0
**PROGENy**	Affected pathways	x1.0
**SLAPenrich**	Affected pathways	x1.0
Expression Atlas	Expression	x0.5
Europe PMC	Text mining	x0.2
PhenoDigm	Animal models	x0.2

Data sources in bold are new data, whereas the remaining sources have been described in our first publication (3) and now contain updated information.

Supplementary Table 2: Number of target-disease associations derived from evidence provided by 16 databases

Database	Association Count*
**Genomics England PanelAPP**	57,262
**PheWAS catalog**	112,544
GWAS catalog	187,351 (*74,146*)
UniProt**	74,990 (*73,999*)
European Variation Archive (germline)	56,495 (*30,411*)
Gene2Phenotype	20,032 (*12,868*)
Cancer Gene Census	79,468 (*35,323*)
IntOGen	11,195 (*9,311*)

European Variation Archive (somatic)	4,268 (*1,284*)
ChEMBL	116,176 (*57,419*)
Reactome (v65)	7,117 (*3,471*)
**PROGENy**	1,172
**SLAPenrich**	134,234
Expression Atlas	407,499 (*889,058*)
Europe PMC	1,533,229 (*1,173,248*)
PhenoDigm	984,801 (*620,329*)

* As per 18.08 release of the Open Targets Platform. Parentheses show the original number (in italics) of association counts reported by (3).

** Including both somatic mutations and genetic variants.

Data sources in bold are new data, whereas the remaining sources have been described in our first publication (3) and now contain updated information.
